# Tablet Keyboard Configuration Affects Performance, Discomfort and Task Difficulty for Thumb Typing in a Two-Handed Grip

**DOI:** 10.1371/journal.pone.0067525

**Published:** 2013-06-26

**Authors:** Matthieu B. Trudeau, Paul J. Catalano, Devin L. Jindrich, Jack T. Dennerlein

**Affiliations:** 1 Harvard School of Public Health, Boston, Massachusetts, United States of America; 2 Dana-Farber Cancer Institute, Boston, Massachusetts, United States of America; 3 California State University San Marcos, San Marcos, California, United States of America; 4 Harvard Medical School, Boston, Massachusetts, United States of America; 5 Northeastern University, Boston, Massachusetts, United States of America; McMaster University, Canada

## Abstract

When holding a tablet computer with two hands, the touch keyboard configuration imposes postural constraints on the user because of the need to simultaneously hold the device and type with the thumbs. Designers have provided users with several possible keyboard configurations (device orientation, keyboard layout and location). However, potential differences in performance, usability and postures among these configurations have not been explored. We hypothesize that (1) the narrower standard keyboard layout in the portrait orientation leads to lower self-reported discomfort and less reach than the landscape orientation; (2) a split keyboard layout results in better overall outcomes compared to the standard layout; and (3) the conventional bottom keyboard location leads to the best outcomes overall compared to other locations. A repeated measures laboratory experiment of 12 tablet owners measured typing speed, discomfort, task difficulty, and thumb/wrist joint postures using an active marker system during typing tasks for different combinations of device orientation (portrait and landscape), keyboard layout (standard and split), and keyboard location (bottom, middle, top). The narrower standard keyboard with the device in the portrait orientation was associated with less discomfort (least squares mean (and S.E.) 2.9±0.6) than the landscape orientation (4.5±0.7). Additionally, the split keyboard decreased the amount of reaching required by the thumb in the landscape orientation as defined by a reduced range of motion and less MCP extension, which may have led to reduced discomfort (2.7±0.6) compared to the standard layout (4.5±0.7). However, typing speed was greater for the standard layout (127±5 char./min.) compared to the split layout (113±4 char./min.) regardless of device orientation and keyboard location. Usage guidelines and designers can incorporate these findings to optimize keyboard design parameters and form factors that promote user performance and usability for thumb interaction.

## Introduction

Tablets were first introduced as an alternative to smartphones and laptop computers to improve the user experience for certain tasks such as browsing the web, email, and playing games [1]. Because of their mobility, ease of use, and low cost compared to desktop and laptop personal computers, tablets are being used for accomplishing the same tasks as computers, with the most frequent task accomplished on tablets being email [2]. Although the tablet’s form factor eliminates the keyboard peripheral, the soft keyboard remains a necessary and frequently used component of tablet interaction.

Because the tablet affords a mobile computing experience, users often hold it with both hands while sitting, standing, or walking. This interaction technique requires a very different posture than interacting with a computer workstation. The user must hold the device while their thumbs simultaneously interact with the touch keyboard. Despite the ergonomic disparity between tablet and computer workstation interaction, the default keyboard configuration on most tablet devices is similar to a computer workstation’s layout, with the keyboard located at the base of the screen. Current tablet keyboard designs may not be appropriate for the postures required by thumb interaction [3], [4].

Numerous studies on computer workstation ergonomics have led to workstation setup recommendations (e.g. ISO-9241 and ANSI/HFES 100 (USA)) and new keyboard designs (i.e., the Microsoft Natural keyboard), while few studies have focused on tablet ergonomics [5], [6], [7]. A split keyboard configuration was recently introduced to tablet operating systems (i.e., Apple Inc.’s iOS 6, Microsoft’s Windows 8) with the goal of reducing thumb reach, but the effectiveness of this keyboard design on performance and usability as well as its ideal location on the screen have not yet been determined. Recent studies investigating the ergonomics of single-handed smartphone interaction suggest that keyboard design parameters such as key size and location on the screen affect tapping performance metrics such as thumb movement time, precision, and Fitts’ motor performance [3], [8], [9]. In addition, Trudeau et al. (2012b) further report that the association between key location and thumb motor performance may be explained by the thumb and wrist postures required to reach the keys. Neutral thumb postures were found to lead to greater motor performances than when the thumb was either flexed or extended [4]. These studies suggest that mobile device keyboard design parameters affect user performance. However, it has not yet been determined whether keyboard design affects performance and usability measures during functional tasks such as thumb typing.

Therefore, we aimed to investigate the effect of tablet keyboard configuration on thumb typing speed, self-reported discomfort, task difficulty, and thumb/wrist postures across configurations for a two-handed grip on a tablet device. We expected that typing speed, self-reported discomfort and task difficulty would vary across keyboard configurations, and that these variations may be due to different thumb/wrist postures required for the simultaneous tasks of reaching the keys and holding the device. More specifically, we tested three hypotheses. First, we expected that the narrower standard keyboard layout in the portrait orientation would require less reach than the landscape orientation and would lead to lower self-reported discomfort than the landscape orientation. Second, we expected that the split keyboard layout would be effective at reducing thumb extension and would result in greater performance and lower self-reported discomfort than the standard keyboard layout. Third, since the bottom keyboard location is currently the default setting on tablet devices, we explored the hypothesis that the bottom keyboard location leads to the greatest typing speed and lowest discomfort and difficulty.

## Methods

### Ethics Statement

Twelve right-handed adults (6 men, 6 women) provided written consent before participating in a repeated measures experiment. The Harvard School of Public Health institutional review board, the Office of Regulatory Affairs and Research Compliance (ORARC), approved all forms and protocols.

### Demographics

Participants mean (±SD) age and right hand length were 29.9±5.1 yrs and 18.9±2.1 cm respectively. All participants were tablet owners, and had no upper extremity musculoskeletal pain or prior hand/finger surgery at the time of the experiment. From a usage questionnaire handed out prior to the experiment, all participants reported that they most frequently used their device with the keyboard in the standard layout and the bottom location, which is the default setting for most tablet models. Half of participants usually operated their device in the portrait orientation and the other half in the landscape orientation.

### Task and Configurations

**Figure 1:**
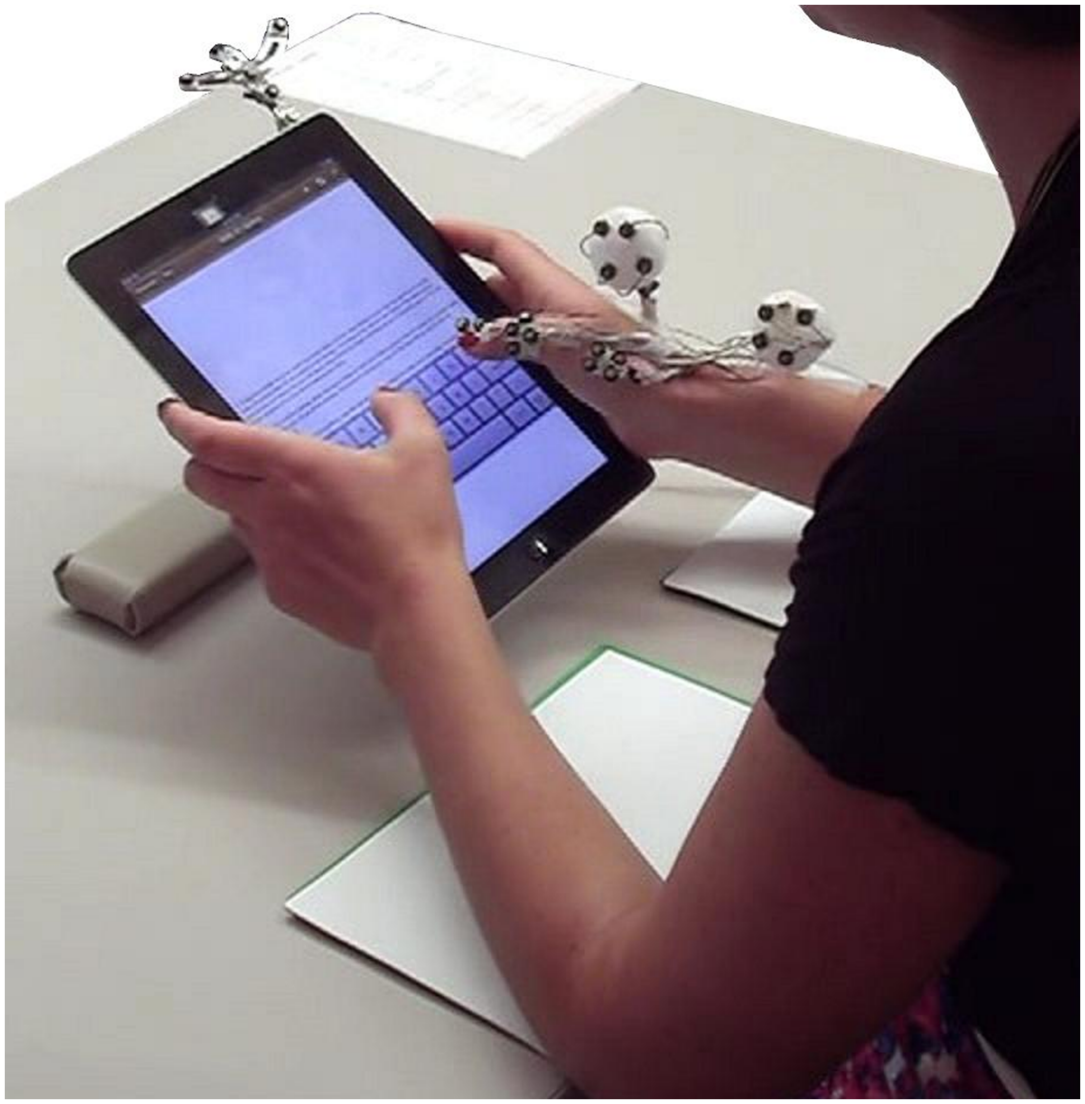
Experimental set-up. The picture illustrates the standard keyboard layout in the middle position with the tablet in the portrait orientation. Markers on the forearm, hand, and thumb segments as well as the tablet computer provided 3D location and orientation of these segments during the experiment. Users were allowed to support their forearms or elbows on the table and to use available padding. The participant pictured here has given written informed consent, as outlined in the PLOS consent form, to publication of their photograph.

Participants accomplished 2-minute typing tasks on an Apple iPad (3^rd^ generation, Apple, Inc.) while holding the device with both hands (Figure 1). This grip configuration required that the participants use only their thumbs to interact with the keys. Participants were seated at a table on a chair without arm supports. After we had provided a summary of the experiment, participants were allowed to adjust the chair height prior to beginning the experiment and to support the device and their elbows or forearms on the table. All nearby light sources were indirect lighting and there was no glare on the tablet’s screen. The tasks involved transcribing a text in a text editing application (Pages, Apple Inc.). Participants read the original text from the tablet screen and transcribed it directly below. Participants were instructed to interact only with the keyboard without touching other parts of the screen (i.e., no scrolling) because we wanted measured postures to exclusively reflect keyboard interaction. The auto-capitalization, auto-correction and check spelling settings were turned off during the trials. We did not alter design parameters that were characteristics of the iPad’s keyboard settings such as key size (i.e., larger in the standard than the split layout) because we considered that these were intrinsic characteristics of the design variables tested on the device. Key size was different across keyboard layout (i.e., key size was larger in the standard than the split layout) and device orientation (key size was larger in the landscape than the portrait orientation with the keyboard in the standard layout), which was an intrinsic characteristic of the operating system and therefore left intact.

**Figure 2:**
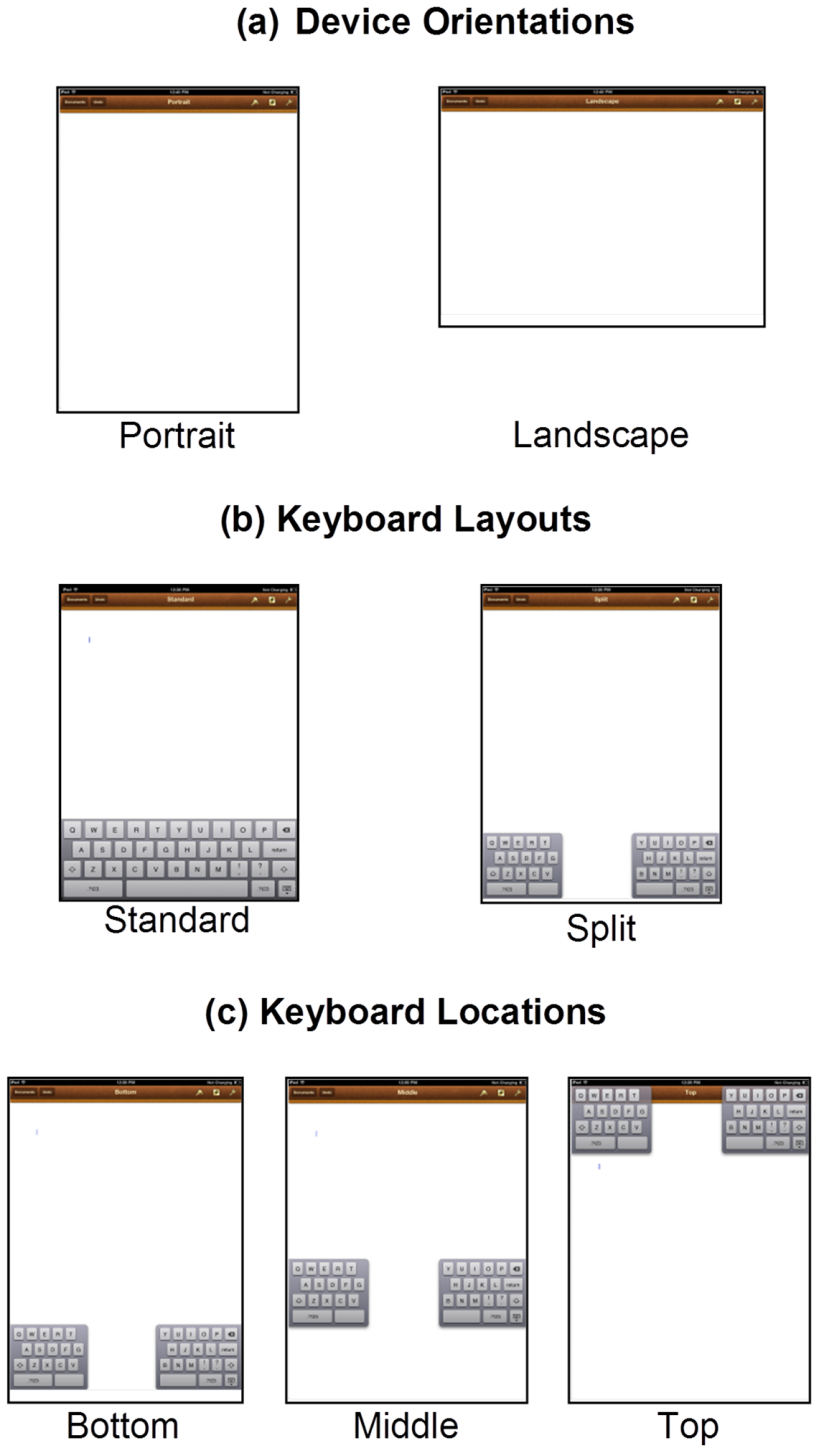
Keyboard configurations. The 11 different configurations involved different combinations of the following 3 independent variables: (a) 2 device orientations, (b) 2 keyboard layouts, and (c) 3 keyboard locations.

Participants accomplished 11 different typing tasks for 11 different texts and keyboard configurations that involved combinations of 3 different independent variables: 2 device orientations (Portrait and Landscape), 2 keyboard layouts (Standard and Split), and 3 keyboard locations (Top, Middle, and Bottom; Figure 2). The design was not a full factorial because the configuration consisting of the combination of the landscape orientation, standard keyboard layout, and middle keyboard location was not included. The amount of screen area available in this configuration was not large enough to see and transcribe the text without scrolling, which the participants were instructed to avoid because: (1) we wanted to determine the effects due strictly to the keyboard design, and (2) scrolling on the screen would have biased the postural measures for that configuration. For the middle keyboard location, the keyboard was positioned at 9.5 cm from the base of the device, which corresponds to the 90th percentile hand breadth for males [10]. This middle keyboard location allowed participants to grip the device on its edge above the bottom corners of the device (Figure 1). The 11 texts were excerpts from The Brothers Grimm fairy tales, and the texts were 350 to 400 characters long, which was long enough that participants did not have time to finish transcribing them within the 2-minute time limit. We randomized the order of the 11 different configurations and texts independently within and across participants.

### Measured Variables

The measured variables were net typing speed (characters/minute), self-reported discomfort, self-reported task difficulty, and hand and thumb joint angles (degrees). We calculated net typing speed as the average gross typing speed minus errors. Prior to the experiment, we told participants that any typographical errors would count against their typing speed. We measured self-reported discomfort and task difficulty from visual analog scales (1–10) collected from a questionnaire that we administered to the participant after every task. Before the experiment, we told participants that self-reported discomfort was a measure of the physical discomfort or pain experienced in any part of the back, neck, arm, hand and/or thumbs while typing, whereas task difficulty was a measure of the cognitive load. We asked participants to justify the self-reported scores through written comments after each trial.

We calculated tablet, thumb, hand, and forearm 3D kinematics measured using an active-marker motion capture system (Optotrak Certus, Northern Digital Inc., Waterloo, Canada). We mounted clusters of three infrared light emitting diodes (IREDs) secured to a rigid plate to the tablet, right forearm, dorsal surface of the hand, first metacarpal and proximal phalange of the thumb, which we considered as rigid body segments, and two IREDs were fixed to the thumb nail (Figure 1). The IRED placement used in this study builds on previous methods for measuring thumb kinematics (i.e., [11], [12], [13], [14], [4]), and accounts for the established degrees of freedom of each joint [15], [16] while minimizing physical and visual obstruction for the participant. We recorded IRED 3D trajectories to a personal computer at 100 Hz, then digitally filtered them through a low-pass, fourth order Butterworth filter with a 10 Hz cutoff frequency. We transformed cluster orientations to describe the anatomical segment location and orientation via the relative location of digitized bony landmarks [17].

We calculated wrist and thumb joint angles from the Euler angles of the rotation matrices describing the orientation of the joint’s distal segment relative to the proximal segment [17]. The first Euler angle rotation was flexion/extension, the second was abduction/adduction and the third was pronation/supination. We expressed joint angles relative to a reference posture in which the longitudinal axes of the forearm and hand were aligned, and the thumb was extended and apposed to the lateral side of the index finger [15]. The calculations assumed the wrist joint has two degrees of freedom (flex/extension, abd/adduction), the thumb’s carpometacarpal (CMC) joint has three degrees of freedom (flex/extension, abd/adduction, pron/supination), the thumb’s metacarpal (MCP) joint has two degrees of freedom (flex/extension, abd/adduction), and the thumb’s interphalangeal (IP) joint has a single degree of freedom (flex/extension).

We calculated median joint angles and joint ranges of motion as metrics to describe hand and thumb posture for each trial. We calculated joint range of motion as the difference between the 90^th^ and the 10^th^ percentile joint angle [18].

### Analysis

To determine the effect of tablet keyboard configurations on thumb typing speed, self-reported discomfort, task difficulty and wrist/thumb median joint angles and range of motion for a two-handed grip on a tablet device, we employed a mixed-effects analysis of variance (ANOVA) model for each of these dependent variables. For each ANOVA model, we included participant as a random effect and all three independent variables (device orientation, keyboard layout, and keyboard location) as fixed effects, as well as all two-way interaction terms. The three-way interaction term (device orientation×keyboard layout×keyboard location) was not significant (two-sided test with a significance level α = 0.05) in every model and therefore it was removed from the final ANOVA models. We also included a “trial order” main effect into each model to account for a possible learning effect. For models in which the main effect “keyboard location” was significant, we used a post hoc Tukey’s Honestly Significant Difference (HSD) test to determine whether differences existed across levels of keyboard location (i.e., top, middle, bottom). All statistical analyses were run using JMP Pro 10 Software (SAS Institute, Cary, NC).

## Results

**Figure 3:**
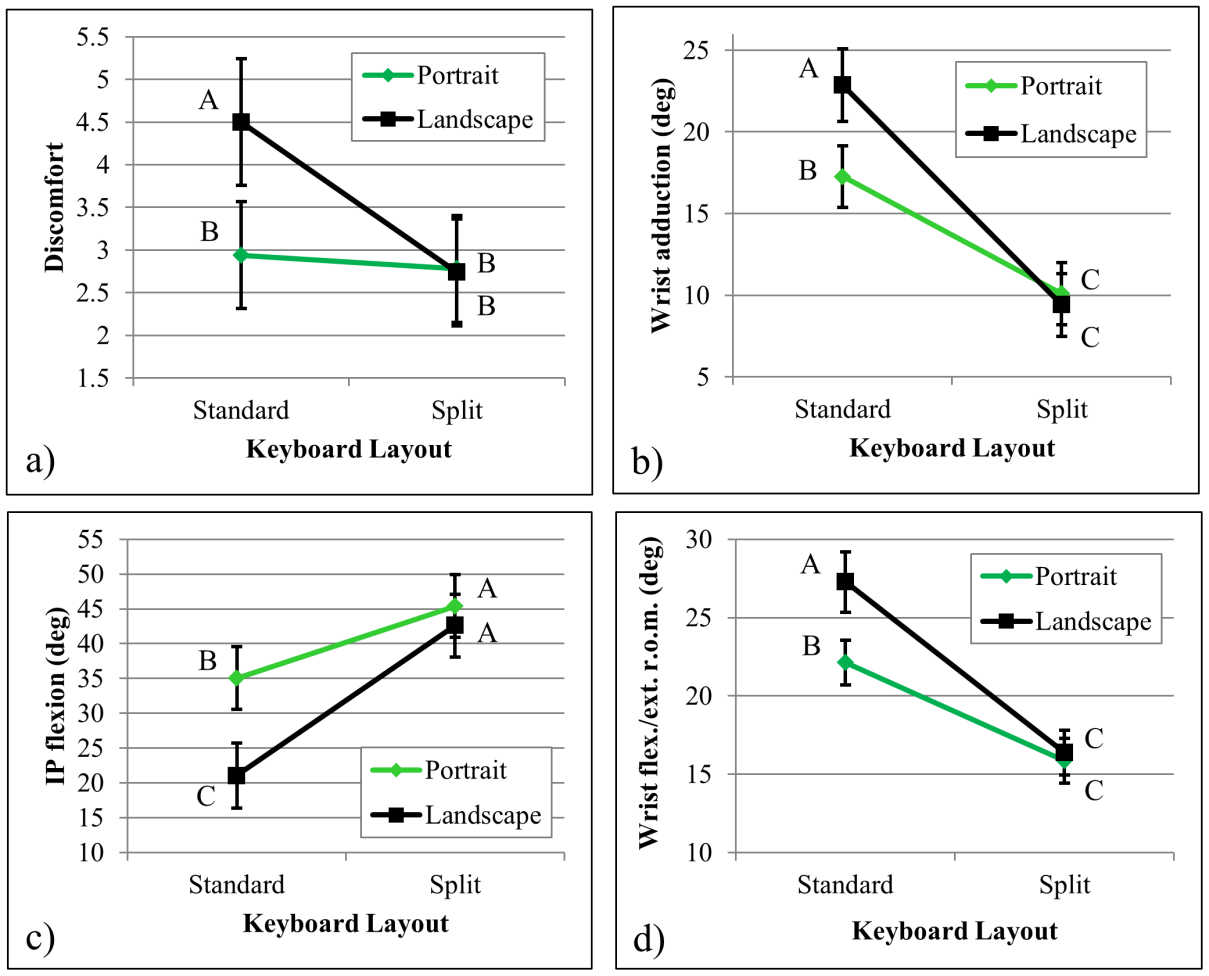
Significant interaction effects between Keyboard Layout and Device Orientation. Least squares (and standard error) values are presented for (a) mean self-reported discomfort across device orientation and keyboard layout, (b) median wrist adduction across device orientation and keyboard layout, (c) median IP flexion across device orientation and keyboard layout, and (d) wrist joint range of motion for the flex./ext. axis across device orientation and keyboard layout. The superscript letters in the figure represent results from the Tukey post-hoc analysis: same letters denote groups without significant differences. Values with different letters are ranked such that A>B>C.

There was a significant interaction effect between device orientation and keyboard layout for self-reported discomfort (F = 5.8, p = 0.018) (Table 1), indicating that discomfort was reduced for the split keyboard layout compared with the standard layout only when the device was in the landscape orientation (2.7±0.6 for the split layout vs. 4.5±0.7 for the standard layout), but not in the portrait orientation (2.8±0.6 for the split layout vs. 2.9±0.6 for the standard layout) (Figure 3a). There were significant interactions between device orientation and keyboard layout for three postural measures as well: wrist adduction (F = 10.4, p = 0.002) (Figure 3b), IP flexion (F = 26.6, p<0.001) (Figure 3c), and wrist range of motion about the flexion/extension axis (F = 4.5, p = 0.037) (Figure 3d). These postural measures were similar across device orientations for the split layout, but they all differed significantly with the keyboard in the standard layout, with the landscape orientation being associated with more wrist adduction, less IP flexion, and more wrist range of motion along the flexion/extension axis. Additionally, median joint angles were not significantly different across device orientations for any other joint degree of freedom (Table 2). Typing speed and self-reported task difficulty were not significantly different across device orientations (F = 1.0, p = 0.314 and F = 2.7, p = 0.102, respectively).

**Table 1:** Least squares mean (and standard error) net typing speed, self-reported discomfort and self-reported task difficulty for main effects Device Orientation, Keyboard Layout, and Keyboard Location, and p-values for interactions.^a^

	Typing speed (char./min.)	Discomfort while typing	Task difficulty
**Device Orientation**			
ANOVA	p** = **0.314	**p = 0.025**	p** = **0.102
Portrait	122 (5)	2.9 (0.6)	3.8 (0.5)
Landscape	119 (5)	3.6 (0.6)	4.3 (0.5)
**Keyboard Layout**			
ANOVA	**p<0.001**	**p = 0.005**	p** = **0.156
Standard	127 (5)	3.7 (0.6)	4.3 (0.5)
Split	113 (4)	2.8 (0.6)	3.8 (0.5)
**Keyboard Location** b			
ANOVA	**p<0.001**	p** = **0.313	**p<0.001**
Top	113 (5)^B^	3.5 (0.6)^A^	4.8 (0.5)^A^
Middle	124 (5)^A^	2.8 (0.7)^A^	3.6 (0.5)^B^
Bottom	123 (5)^A^	3.4 (0.6)^A^	3.7 (0.5)^B^
**Interactions**			
Orientation×Layout	p** = **0.421	**p = 0.018**	p** = **0.940
Orientation×Location	p** = **0.102	p** = **0.720	p** = **0.246
Layout×Location	p** = **0.717	p** = **0.487	p** = **0.343

a Statistically significant ANOVA results are in bold.

b The superscript letters in the table represent results from the Tukey post-hoc analysis: same letters denote groups without significant differences. Values with different letters are ranked such that A>B.

**Table 2:** Least squares median (and standard error) joint angles (^o^) for main effects Device Orientation, Keyboard Layout, and Keyboard Location, and p-values for interactions.a,b

	Wrist		CMC			MCP		IP
	Extension (°)	Adduction (°)	Extension (°)	Abduction (°)	Pronation (°)	Extension (°)	Abduction (°)	Flexion (°)
**Device Orientation**								
ANOVA	p = 0.855	**p = 0.012**	p = 0.778	p = 0.093	p = 0.721	p = 0.663	p = 0.859	**p<0.001**
Portrait	13 (4)	14 (2)	7 (2)	9 (2)	2 (2)	4 (2)	16 (2)	40 (4)
Landscape	13 (4)	16 (2)	7 (2)	10 (2)	1 (2)	4 (3)	16 (2)	32 (5)
**Keyboard Layout**								
ANOVA	**p = 0.002**	**p<0.001**	p = 0.901	**p = 0.001**	p = 0.801	**p<0.001**	p = 0.184	**p<0.001**
Standard	16 (4)	20 (2)	7 (2)	11 (2)	2 (2)	8 (3)	15 (2)	28 (5)
Split	11 (4)	10 (2)	7 (2)	9 (2)	1 (2)	0 (2)	16 (2)	44 (4)
**Keyboard Location** c								
ANOVA	**p = 0.036**	**p<0.001**	**p<0.001**	**p<0.001**	**p<0.001**	p = 0.081	**p = 0.003**	p = 0.156
Top	11 (4)^B^	18 (2)^A^	10 (2)^A^	9 (2)^B^	−1 (2)^B^	6 (3)^A^	14 (1)^B^	35 (4)^A^
Middle	16 (4)^A^	16 (2)^A^	8 (3)^A^	9 (2)^B^	1 (3)^B^	4 (3)^A^	16 (2)^A,B^	37 (5)^A^
Bottom	13 (4)^A,B^	11 (2)^B^	5 (2)^B^	12 (2)^A^	4 (2)^A^	2 (3)^A^	17 (2)^A^	37 (4)^A^
**Interactions**								
Orientation×Layout	p = 0.732	**p = 0.002**	p = 0.531	p = 0.336	p = 0.976	p = 0.836	p = 0.871	**p<0.001**
Orientation×Location	p = 0.908	p = 0.098	p = 0.836	p = 0.675	p = 0.884	p = 0.514	p = 0.642	p = 0.240
Layout×Location	**p = 0.003**	p = 0.168	**p<0.001**	p = 0.311	**p<0.001**	p = 0.526	p = 0.607	p = 0.120

a Statistically significant ANOVA results are in bold.

b Joint angles were expressed relative to a reference posture where the longitudinal axes of the forearm and hand were aligned, and the thumb was straight and apposed to the lateral side of the index finger.

c The superscript letters in the table represent results from the Tukey post-hoc analysis: same letters denote groups without significant differences. Values with different letters are ranked such that A>B.

Net typing speed was significantly greater in the standard layout (127±5 char/min) compared to the split layout (113±4 char/min) (F = 21.7, p<0.001) (Table 1). The MCP joint was more extended and the CMC joint more abducted in the standard layout (MCP extension median angle (S.E.) 8±3° for the standard layout compared to 0±2° for the split layout, and CMC abduction median angle (S.E.) 11±2° for the standard layout compared to 9±2° for the split layout). Additionally, joint ranges of motion were all greater for the standard keyboard layout compared to the split layout (Table 3), except for CMC joint abduction/adduction and supination/pronation, for which range of motions were not significantly different across layouts.

**Table 3:** Least squares joint range of motion (and standard error) for main effects Device Orientation, Keyboard Layout, and Keyboard Location, and p-values for interactions.a,b

	Wrist		CMC			MCP		IP
	Flex./Ext. (°)	Ab./Add. (°)	Flex./Ext. (°)	Ab./Add. (°)	Sup./Pron. (°)	Flex./Ext. (°)	Ab./Add. (°)	Flex./Ext. (°)
**Device Orientation**								
ANOVA	**p = 0.011**	p = 0.803	p = 0.583	**p = 0.013**	p = 0.121	p = 0.846	p = 0.721	p = 0.713
Portrait	20 (1)	9 (1)	14 (1)	13 (1)	17 (2)	19 (2)	16 (1)	28 (2)
Landscape	22 (1)	9 (1)	14 (1)	12 (1)	16 (2)	19 (2)	16 (1)	28 (2)
**Keyboard Layout**								
ANOVA	**p<0.001**	**p<0.001**	**p<0.001**	p = 0.341	p = 0.988	**p<0.001**	**p = 0.003**	**p<0.001**
Standard	25 (1)	10 (1)	15 (1)	12 (1)	17 (2)	21 (2)	17 (1)	31 (2)
Split	16 (1)	7 (1)	13 (1)	13 (1)	17 (2)	17 (2)	15 (1)	24 (2)
**Keyboard Location** c								
ANOVA	p = 0.566	p = 0.354	**p = 0.014**	**p = 0.024**	**p = 0.010**	p = 0.624	p = 0.321	**p = 0.002**
Top	21 (1)^A^	9 (1)^A^	13 (1)^B^	14 (1)^A^	16 (2)^B^	19 (2)^A^	16 (1)	26 (2)^B^
Middle	19 (2)^A^	8 (1)^A^	14 (1)^A,B^	12 (1)^A,B^	15 (2)^B^	20 (2)^A^	16 (1)	27 (2)^A,B^
Bottom	21 (1)^A^	9 (1)^A^	15 (1)^A^	12 (1)^B^	18 (2)^A^	19 (2)^A^	15 (1)	30 (2)^A^
**Interactions**								
Orientation×Layout	**p = 0.037**	p = 0.823	p = 0.101	p = 0.129	p = 0.152	p = 0.652	p = 0.640	p = 0.233
Orientation×Location	p = 0.562	p = 0.217	p = 0.800	p = 0.480	p = 0.366	p = 0.639	p = 0.505	p = 0.685
Layout×Location	p = 0.703	p = 0.116	p = 0.252	p = 0.564	**p = 0.008**	p = 0.513	p = 0.141	p = 0.247

a Statistically significant ANOVA results are in bold.

b Range of motion was calculated from the difference between the 90^th^ and 10^th^ percentile joint angles within each trial.

c The superscript letters in the table represent results from the Tukey post-hoc analysis: same letters denote groups without significant differences. Values with different letters are ranked such that A>B.

Participants typed significantly slower (F = 7.8, p<0.001) and reported significantly more difficulty (F = 7.7, p<0.001) in the top location compared to the middle and bottom locations (Table 1). Although participants reported feeling less discomfort for the middle keyboard location (2.8±0.7) compared to both the top (3.5±0.6) and bottom locations (3.4±0.6), the difference was not statistically significant (F = 1.17, p = 0.313). The wrist was less adducted in the bottom location (11±2°) than in the middle (16±2°) and top (18±2°) locations, and the CMC and MCP joints were more abducted in the bottom location (median angles (S.E.) 12±2° for the bottom, 9±2° for middle and top locations for the CMC joint, and median angles (S.E.) 17±2° for the bottom, 16±2° for middle, 14±1° for the top location for the MCP joint).

**Figure 4:**
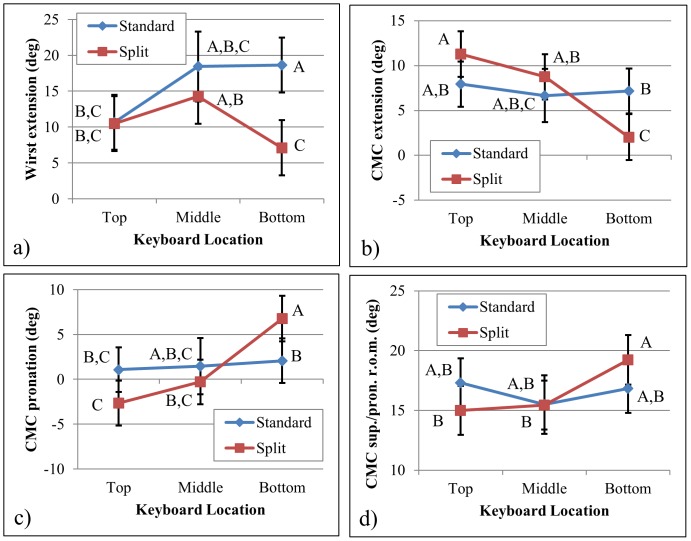
Significant interaction effects between Keyboard Location and Keyboard Layout. Least squares (and standard error) values are presented for (a) mean median wrist extension across keyboard location and keyboard layout, (b) median CMC extension across keyboard location and keyboard layout, (c) median CMC pronation across keyboard location and keyboard layout, and (d) CMC joint range of motion for the sup./pron. axis across keyboard location and keyboard layout. The superscript letters in the figure represent results from the Tukey post-hoc analysis: same letters denote groups without significant differences. Values with different letters are ranked such that A>B>C.

There were significant interaction effects between keyboard location and layout for four of the postural measures: wrist median extension (F = 6.1, p = 0.003), CMC median extension (F = 13.6, p<0.001), CMC median pronation (F = 8.7, p<0.001), and CMC range of motion about the sup./pronation axis (F = 5.1, p = 0.008) (Figure 4). No clear patterns emerged from plotting these interactions, except that the CMC appeared to be less extended and more pronated as the keyboard was located lower down on the device in the split keyboard layout (Figures 4b and 4c, respectively). Additionally, the wrist was significantly less extended for the split layout than the standard layout for the bottom location (7±4° for the split and 19±4° for the standard layout) but not for the top and middle locations (Figure 4a).

## Discussion

The aim of this study was to determine whether keyboard configuration affects thumb typing speed, self-reported discomfort, task difficulty, and thumb/wrist postures. We determined that thumb typing speed, self-reported discomfort, task difficulty, and median and range of motion postures all vary across keyboard configurations, with the portrait orientation and middle keyboard location leading to the greatest typing speed and least discomfort and task difficulty. Measured thumb and wrist posture results suggest that different keyboard configurations require different holding and reaching requirements of the thumb, with greater reach and awkward postures possibly having a negative effect on user performance and usability.

The results need to be considered within the context of the study’s limitations. First, we only considered the support condition in which the device is held with a two-handed grip. Other support conditions such as finger typing with the device supported on a desk, with or without a case, in one hand, or using an external keyboard, could potentially yield different results. A next step would be to compare the results from this study with conditions in which the user holds the device with one hand while interacting with the other, or with the device fully supported on a desk or the lap. These data could be used to establish usage recommendations and for designing tablet hardware and software that favor the particular technique that is found to be associated with superior performance and usability, and that favors a more neutral posture. Additionally, certain design parameters such as key size and shape changed with the keyboard configuration and may have affected the results. We considered that these were intrinsic characteristics of the operating system. Therefore, the results may only be generalizable to the iPad’s iOS 6 and operating systems in which the keyboard designs are similar.

Another limitation to this study is that we instructed participants to transcribe a text that they read from the mobile device’s screen, which may require a greater cognitive load than composing a text from memory. However, we expected the cognitive load due to transcribing the text to be consistent across keyboard configurations because the task was consistent across configurations. Moreover, transcribing the text from the screen required that the participant maintain visual access to the screen, which we determined to be an intrinsic characteristic of the task. Next, since all the participants reported most frequent use of their own device with the keyboard in the standard layout and bottom location, it is possible that the task difficulty results may be biased toward favoring these conditions. For the keyboard layout variable, the results are conservative in the context of this potential bias because task difficulty was found to be slightly greater for the standard layout (i.e., the potential experience bias pulls this effect toward the Null). With respect to the keyboard location variable, removal of a potential experience bias would imply greater reported discomfort for the bottom location, which would favor the middle and top locations.

Next, the study design was unbalanced because the landscape, standard, middle configuration was not included. We did not test the landscape/middle/standard configuration because the limited screen area available for displaying the text in this configuration would have required that the participant scroll through the text by touching a finger to the screen, which would have biased the postural measures for this configuration. Our instructions for the participant were to interact strictly with the keyboard because we were specifically interested in determining the performance, discomfort, difficulty and postures that were a direct result of the keyboard configuration demands. Learning presents another potential source of bias due to the fact that most participants did not have extensive experience typing with their thumbs under the different keyboard configurations. We accounted for this bias in the study design by randomizing the presentation of the configurations for each participant, and in the statistical analysis by including a “trial order” parameter as a main effect in the ANOVA models. Lastly, trunk and lower body posture were standardized by having the participants sit at a desk. Although this setting may not be generalizable to common usage across all participants, we do not expect that the results would vary substantially across configurations in a different setting.

Discomfort was reduced when users typed on the standard keyboard layout with the device in the portrait orientation compared to the landscape orientation (Figure 3a), supporting hypothesis 1. However, the difference in discomfort did not impact performance across orientations, which was only affected by the keyboard’s layout and location (Table 1). The interaction plots in Figures 3c and 3d further support the first hypothesis that the reduced discomfort in the portrait orientation may have been due to a reduced amount of reach required by the narrower keyboard, with the IP joint being more flexed and the wrist range of motion being reduced for the portrait compared to the landscape orientation with the device in the standard keyboard layout. The significant interaction effect between device orientation and keyboard layout on self-reported discomfort (Figure 3a) further indicates that the split keyboard layout was effective at reducing discomfort in the landscape orientation, but not in the portrait orientation, which partially supports hypothesis 2. Decreased wrist and thumb range of motion (Table 3) for the split compared to the standard layout suggests that the split keyboard was effective at decreasing the amount of reaching by the thumb, which further supports hypothesis 2. However, the decreased reach requirements did not result in greater performance, which was unexpected because we assumed that reduced discomfort would translate into greater performance. Increased typing speed in the standard layout may have resulted from the larger key size for the standard layout [9]. Additionally, several participants reported that the split keyboard layout required greater concentration because of having to look left and right at both halves of the keyboard (i.e., a non-continuous zone), which may have increased the cognitive load [19]. The split keyboard layout further required that participants retract their thumbs in a clawing posture, possibly to prevent visual obstruction to the keys. Task difficulty did not vary across keyboard layouts, which was unexpected especially given that all the participants reported being more familiar with the standard layout.

Variations in thumb and wrist postures across keyboard locations suggest that each keyboard location constrained the users to hold the device in a way that affected typing speed and task difficulty. The top location led to the worst typing speed and task difficulty compared to the other locations, and the middle and bottom locations were associated with similar typing speed and difficulty results. These results partially support hypothesis 3 that the bottom location would yield the best results. However, discomfort was lowest for the middle location (although not statistically significant). With the keyboard in the middle location, the user could grip the device close to its transverse axis, which allowed them to rest the device against the palm of their fingers in a comfortable posture without the need to exert additional effort to support the device while typing. To reach the keys in the bottom location, the user was constrained to holding the device in the bottom corners. This placed the hands far from the device’s center of mass, generating a moment of force from the increased moment arm that the hand and finger muscles were required to support using a supinated forearm to support the back of the device while the thumb was in opposition to reach the keys. Postural measures were consistent with this behavior, with less CMC extension (median angle (S.E.) 5±2°) and greater pronation (median angle (S.E.) 4±2°) for the bottom keyboard location compared to the top (median angle (S.E.) 10±2° CMC extension and −1±2° CMC pronation) (Table 2, and Figures 4b and 4c). Some participants reported discomfort from the sharp corners of the device digging into their palms, which may have contributed to the higher discomfort scores reported for the bottom location compared to the middle. When the keyboard was in the top location, participants gripped the device at the top edge, which required that they adduct their wrist (median angle (S.E.) 18±2°) to maintain a perpendicular viewing angle with the screen. From an anatomical perspective, extreme wrist adduction as defined by a posture approaching the joint’s motion limit places the thumb’s extrinsic flexor muscles in a tensed state, increasing the thumb joint’s passive forces [20], [21]. This may have been a factor contributing to the greatest self-reported discomfort and lowest typing speed results for the top location. From participant comments, the greater reported task difficulty for the top keyboard location was likely a consequence of the unusual task of having to look at the text below the keyboard (as opposed to above) while typing. The more familiar layout involving the text above the keyboard (i.e., in the case of the bottom and middle keyboard locations) led to lower task difficulties, partially supporting hypothesis 3.

This study’s results relate to usage guidelines and design recommendations for two-handed grip. First, when gripping the tablet with two hands, using a split keyboard layout may reduce discomfort from reaching with the thumbs but only when holding the device in the landscape orientation. If the device is used in the portrait orientation, then the standard keyboard layout is best because it may lead to greater performance compared to the split layout. A middle keyboard location could improve typing performance compared to the top location and reduce discomfort from the sharp corners at the bottom of the device. There is a compromise between keyboard vertical location and the ability to see the page/text, but this can be partially mitigated if the split keyboard layout allows the user to see the text between both halves of the keyboard such as was the case with the operating system tested (Apple Inc.’s iOS 6). Designers could also consider modifying the split keyboard layout to better match the thumb’s range of motion [4], and improving the grip by adding friction to the back side of the device. Lastly, it is important for users to be aware of the ability to customize keyboard settings as most of the participants in this study were unaware of the keyboard configuration options despite owning the same device and operating system as the one used in the study.

### Conclusion

Our results demonstrate that, for a two-handed grip on a tablet, thumb typing speed, self-reported discomfort, task difficulty and thumb/wrist postures vary across keyboard configurations. With the keyboard in the standard layout, participants reported feeling less discomfort when typing with the device in the portrait orientation compared to the landscape orientation. Additionally, the split keyboard layout was effective at reducing discomfort and reach when the device was in the landscape orientation, but not in the portrait orientation. Discomfort was lowest with the keyboard in the middle location, which required participants to grip the device closer to its transverse axis. Based on these results, a tablet keyboard designed for thumb interaction can potentially improve performance if it accounts for the grip and reach requirements imposed on the user by the locations of the keys with respect to the device’s form factor. These results could be used by designers to determine thumb keyboard design parameters and default settings as well as device form factors that promote user performance and usability for tablet thumb interaction.
